# Appendiceal neuroendocrine tumors: a case series and literature review

**DOI:** 10.1093/jscr/rjaf237

**Published:** 2025-04-23

**Authors:** Mohammed N AlAli, Salman T AlWadani, Sadiq M Amer, Mohamed S Essa, Maha AlAmodi, Hussain M AlHassan, Arwa F Alrasheed, Ahlam A Alsulaiman, Saud K Aldeghaither

**Affiliations:** Department of Surgery, Prince Mohammed bin Abdulaziz Hospital, Ministry of Health, Riyadh, Saudi Arabia; Department of Surgery, Prince Mohammed bin Abdulaziz Hospital, Ministry of Health, Riyadh, Saudi Arabia; Department of Pathology, Prince Mohammed bin Abdulaziz Hospital, Ministry of Health, Riyadh, Saudi Arabia; Department of Surgery, Prince Mohammed bin Abdulaziz Hospital, Ministry of Health, Riyadh, Saudi Arabia; General Surgery Department, Faculty of Medicine, Benha University, Benha Egypt; Department of Surgery, College of Medicine, King Khalid University, Abha, Saudi Arabia; College of Medicine, Dar Al Uloom University, Riyadh, Saudi Arabia; Department of Surgery, Prince Mohammed bin Abdulaziz Hospital, Ministry of Health, Riyadh, Saudi Arabia; Department of Surgery, Ad Diriyah Hospital, Ministry of Health, Riyadh, Saudi Arabia; Department of Surgery, Prince Mohammed bin Abdulaziz Hospital, Ministry of Health, Riyadh, Saudi Arabia

**Keywords:** appendiceal neuroendocrine tumors, appendix, neuroendocrine tumor, hemicolectomy, appendectomy, abdominal pain

## Abstract

Appendiceal neuroendocrine tumors (ANETs) are rare gastrointestinal malignancies that are often diagnosed incidentally during or after surgery for suspected appendicitis, presenting significant diagnostic challenges. Existing studies primarily focus on the epidemiology and management of ANETs but lack comprehensive analyses of diagnostic limitations and treatment outcomes. This study presents five cases of ANETs, emphasizing the ongoing challenges in preoperative diagnosis. Consistent with the medical literature, tumors smaller than 1 cm were effectively managed with appendectomy, whereas a larger tumor with high-risk features necessitated right hemicolectomy. Preoperative imaging consistently failed to identify the tumors, underscoring its limitations in detecting neoplastic causes of appendicitis. These findings highlight the need for larger-scale studies, the development of advanced imaging techniques—particularly with the integration of artificial intelligence—and standardized follow-up protocols for high-risk cases.

## Introduction

Acute appendicitis is the most commonly encountered surgical emergency worldwide [1]. However, its clinical presentation often overlaps with other appendicular pathologies, making accurate preoperative diagnosis challenging for medical teams [2].

One such condition complicating diagnosis is the appendiceal neuroendocrine tumor (ANET), a rare subtype of neuroendocrine tumor (NET) that accounts for ~2% of all malignancies [3]. ANETs, the most common type of gastrointestinal NET, typically present with vague and nonspecific symptoms resembling acute appendicitis. Consequently, these tumors are often discovered incidentally during appendectomies, colonoscopies [4], or through postoperative pathological evaluations, further compounding diagnostic challenges [5].

Despite their rarity, ANETs have been reported in ~1% of appendectomies across various studies [6, 7], although some reports indicate variations in prevalence. For instance, a retrospective review of 1110 appendectomy cases found that 25 cases were appendiceal neoplasms, and among them, 12% were neuroendocrine tumors, equating to ~0.27% of all appendectomies in that study cohort [8]. However, comprehensive data on ANETs remain limited, particularly in the context of larger-scale studies [8]. This scarcity of data hinders the understanding of their true prevalence, clinical presentation, and optimal management strategies.

Compared to NETs of other primary sites and other appendiceal malignancies such as adenocarcinoma, ANETs tend to occur in younger individuals, with an average age of diagnosis ranging from 38 to 51 years. Additionally, a significant female predominance has been reported, with a 2:1 ratio observed in several Western studies [9].

In light of these factors, we present five incidental cases of appendiceal neuroendocrine tumors, all of which were initially diagnosed as acute appendicitis based on clinical presentation and imaging findings.

## Case presentation

### Case 1

**Figure 1:**
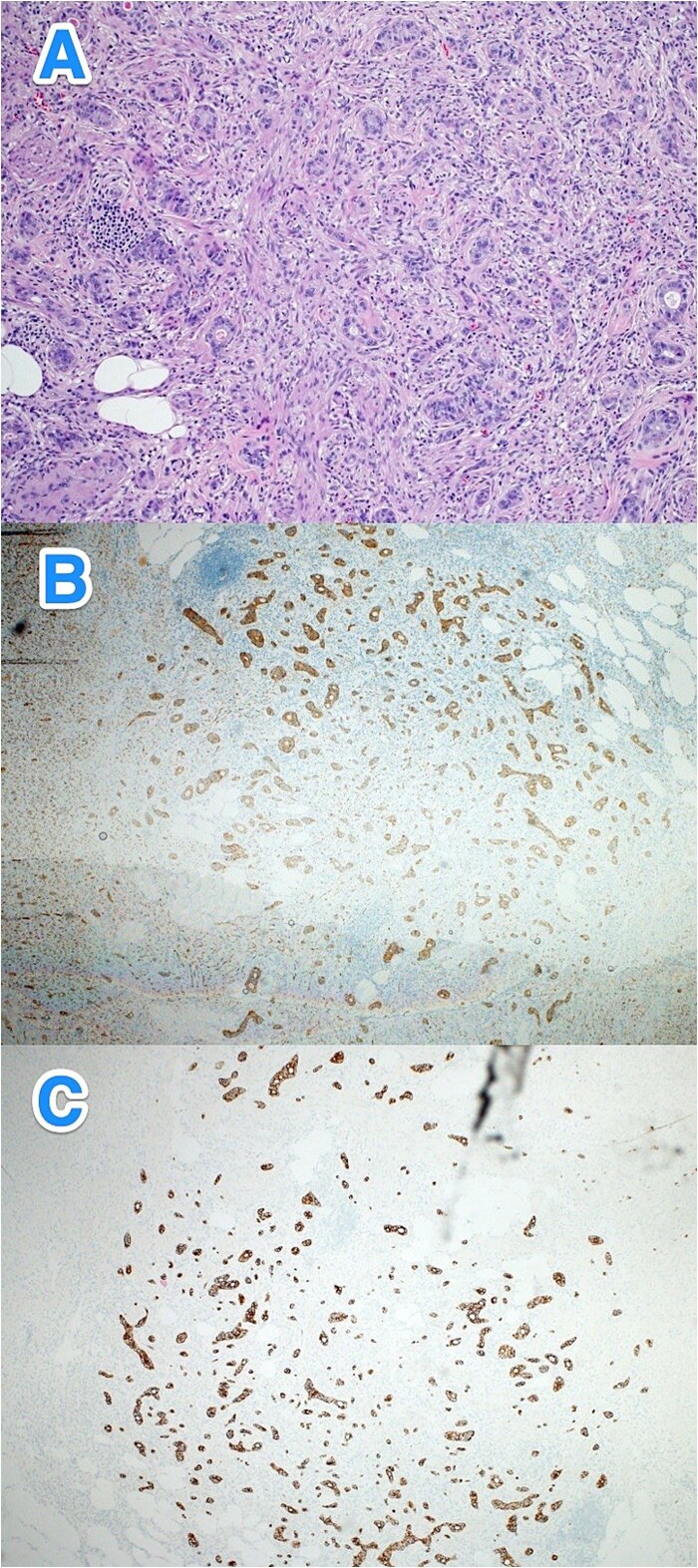
Light microscopy photographs of the appendix show (A) a well-differentiated grade 1 neuroendocrine tumor invading through the muscularis propria into the subserosa, located in the distal half of the appendix. The tumor cells are diffusely positive for (B) synaptophysin and (C) panCK. The Ki-67 labeling index is < 1% (hematoxylin and eosin stain; 2×).

A 28-year-old male with no known past medical or surgical history presented to the emergency department (ED) with a one-day history of symptoms consistent with appendicitis. On examination, his vital signs were normal, but he had right lower quadrant (RLQ) tenderness and rebound tenderness. His white blood cell (WBC) count was 12.9, while other laboratory results were within normal limits. A computed tomography (CT) scan of the abdomen revealed early acute appendicitis. The patient subsequently underwent an open appendectomy, and his postoperative course was uneventful. Histopathological examination revealed a well-differentiated NET (WHO grade I, pTNM: T1Nx) located in the distal half of the appendix (Fig. 1A–C).

### Case 2

**Figure 2:**
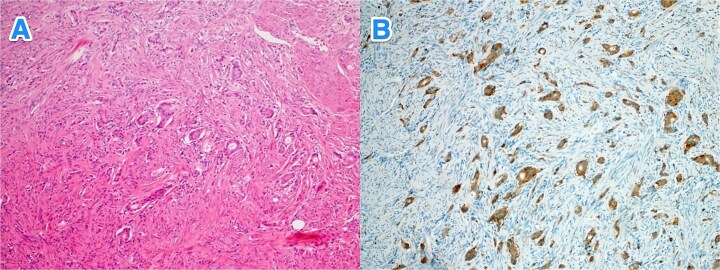
Light microscopy photographs of the appendix show (A) a well-differentiated grade 1 neuroendocrine tumor invading from the submucosa into the muscularis propria, located in the distal half of the appendix (tip). The tumor cells are diffusely positive for (B) synaptophysin (hematoxylin and eosin stain; 2×).

A 37-year-old male with no known past medical or surgical history presented to the ED with a typical history of appendicitis that started earlier that morning. On examination, his vital signs were normal, but he exhibited RLQ tenderness and rebound tenderness. His WBC count was 17.3, while other laboratory results were within normal limits. CT scan of the abdomen confirmed acute appendicitis. During an open appendectomy, the appendix was found to measure 2 cm in diameter, and the postoperative course was uneventful. Histopathological examination revealed a well-differentiated NET (WHO grade I, pTNM: T1Nx) in the proximal half of the appendix. The remaining appendix showed evidence of early acute appendicitis (Fig. 2A–B).

### Case 3

**Figure 3:**
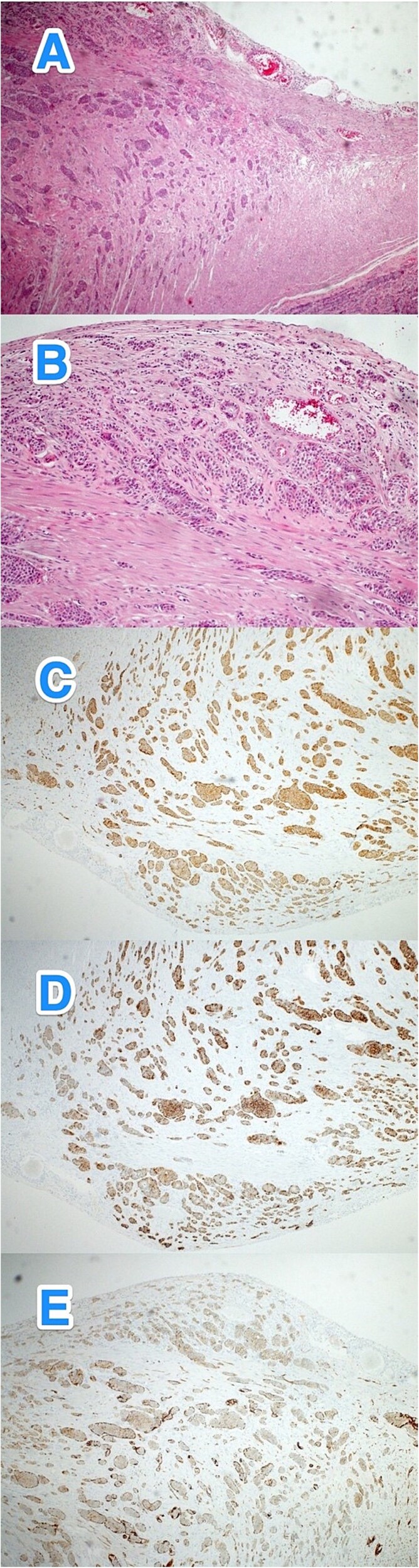
Light microscopy photographs of the appendix show (A, B) a well-differentiated grade 1 neuroendocrine tumor invading from the muscularis propria into the subserosa, located in the distal half of the appendix (tip). The tumor cells are positive for (C) synaptophysin but negative for (D) chromogranin A and (E) CD56. The Ki-67 labeling index is less than 3% (hematoxylin and eosin stain; 2×).

A 43-year-old male with a medical history of bronchial asthma, hypertension, and percutaneous coronary intervention 5 years ago (for myocardial infarction) presented to the ED with a 2-day history of symptoms typical of appendicitis. On examination, his vital signs were normal, but he had RLQ tenderness and rebound tenderness, as well as an incarcerated umbilical hernia. His WBC count was 11.5, and other laboratory results were within normal limits. A CT scan of the abdomen revealed acute appendicitis with a normal-appearing bowel within the umbilical hernia. The patient underwent diagnostic laparoscopy, which revealed pus in the abdomen, an inflamed appendix, and severe inflammation near the appendicular base and ileocecal valve. Due to these findings, the procedure was converted to a laparotomy with ileocecal resection, anastomosis, and abdominal washout. The postoperative course was uneventful. Histopathological examination revealed a well-differentiated NET (WHO grade I, pTNM: T3Nx) with evidence of lymphovascular and perineural invasion. Creatine kinase (CK) was 242. The patient was referred to a higher center for further assessment and management (Fig. 3A–E).

### Case 4

**Figure 4:**
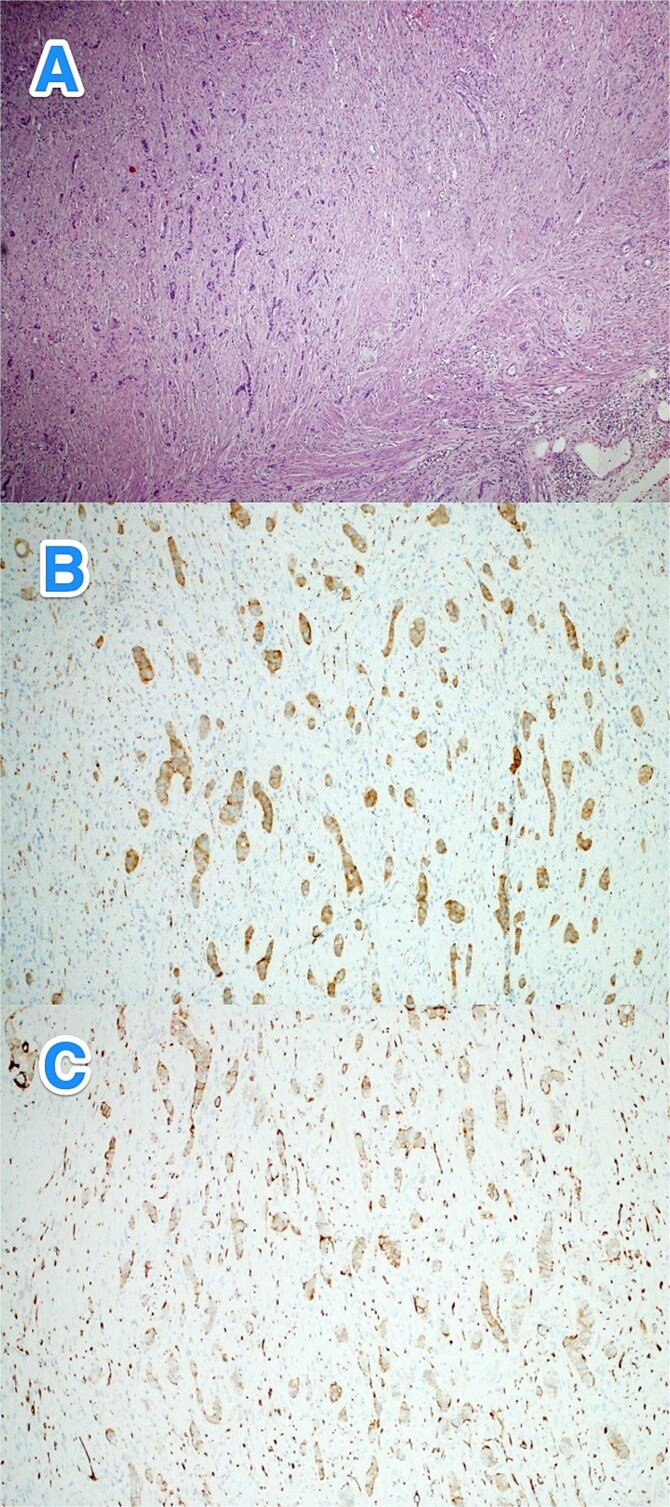
Light microscopy photographs of the appendix show (A) a well-differentiated grade 1 neuroendocrine tumor invading from the muscularis propria into the mesoappendiceal fat, located in the distal half of the appendix. The tumor cells are positive for (B) synaptophysin and (C) CD56 (hematoxylin and eosin stain; 2×).

A 36-year-old female with no known past medical or surgical history presented to the ED with a four-day history of symptoms consistent with typical appendicitis. She had normal vital signs, with RLQ tenderness, rebound tenderness, and a positive Rovsing's sign. Her WBC count was 24, while other laboratory results were within normal limits. CT scan of the abdomen revealed complicated acute appendicitis. An open appendectomy identified a perforated appendix with a pelvic abscess. The postoperative course was uneventful. Histopathological examination revealed a well-differentiated NET (WHO grade I, pTNM: T3Nx) in the distal appendix, with tumor invasion into the mesoappendiceal fat. The remainder of the appendix exhibited suppurative appendicitis with periappendicitis. Consequently, the patient underwent a laparoscopic right hemicolectomy, which confirmed no residual NET or other abnormalities (Fig. 4A–C).

### Case 5

**Figure 5:**
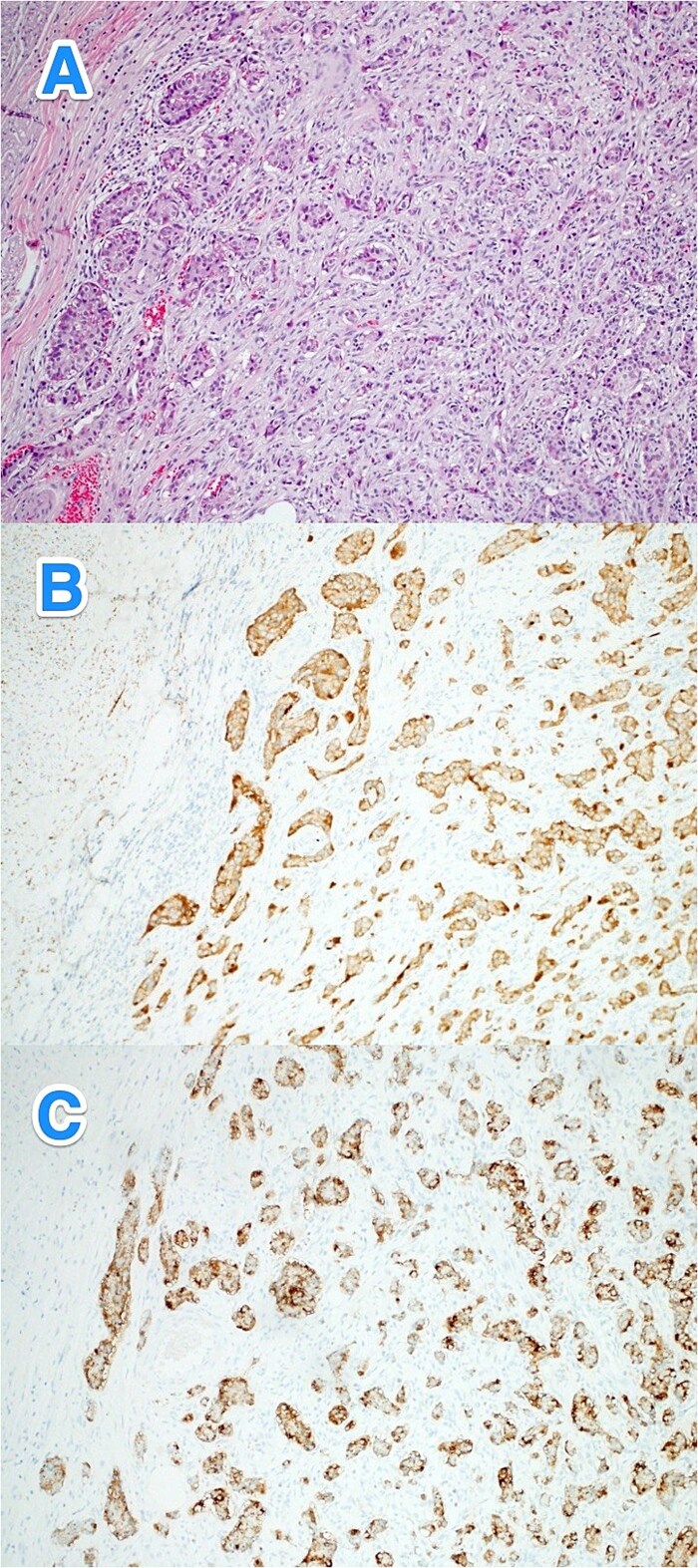
Light microscopy photographs of the appendix show (A) a well-differentiated grade 1 neuroendocrine tumor invading from the submucosa into the muscularis propria, located in the distal half of the appendix. The tumor cells are strongly positive for (B) synaptophysin and (C) chromogranin. The Ki-67 labeling index is less than 2% (hematoxylin and eosin stain; 2×).

A 30-year-old male with no known past medical or surgical history presented to the ED with a 1-day history of severe epigastric and umbilical tenderness, without rebound tenderness. His vital signs were normal, except for elevated blood pressure at 163/97. His WBC count was 16.1, and other laboratory results were unremarkable. A CT scan of the abdomen indicated complicated acute appendicitis. An open appendectomy confirmed the diagnosis of appendicitis. The postoperative course was uneventful. Histopathological examination revealed a well-differentiated NET (WHO grade I, pTNM: T3Nx) in the distal appendix, with tumor invasion into the subserosa and visceral peritoneum. Lymphovascular and perineural invasion were also noted. The remainder of the appendix showed acute appendicitis. The patient was referred to a higher center for further treatment (Fig. 5A–C).

## Discussion

In this case series, there was a male predominance, with four of the five patients being male, despite most studies reporting a higher prevalence of ANETs in females [5]. The patients’ ages ranged from 28 to 43 years, consistent with the typical age range for ANET diagnosis, generally between 38 and 51 years [9].

Almost all patients presented with classic symptoms of acute appendicitis, with no significant differences in clinical presentation between genders. Preoperative imaging uniformly suggested acute appendicitis, with no specific findings indicating an underlying tumor. The diagnosis of ANETs was made postoperatively through histopathological examination. A postoperative review of imaging by radiologists revealed no signs suggesting the presence of tumors. This underscores the limitations of preoperative imaging, as a study of 5224 appendectomy cases found that CT scans identified suspected tumors in only 3.4% of cases with invasive tumors and 16% of cases with noninvasive tumors [10]. These findings highlight the low sensitivity of CT imaging in detecting neoplastic causes of acute appendicitis.

However, advances in artificial intelligence (AI) hold promise for improving the accuracy of medical imaging in detecting appendiceal neuroendocrine tumors. Recent studies have demonstrated AI’s potential in improving the diagnostic accuracy of gastrointestinal malignancies, particularly through the use of convolutional neural networks for radiologic image interpretation [11, 12]. AI-driven algorithms trained on large datasets have been successfully integrated into clinical workflows for detecting pancreatic and colorectal neuroendocrine tumors with improved sensitivity compared to conventional imaging alone [13, 14].

The most frequent location of ANETs is the tip of the appendix (~70%), with only 5%–20% of cases arising in the mid-appendix and <10% occurring at the base, making obstruction of the appendix rare [9]. Tumor size is a critical determinant of prognosis and guides management decisions [3]. ANETs are typically grade 1 (G1) or grade 2 (G2) NETs according to the 2010 WHO classification (i.e. Ki-67 index <20%), and more than 95% are <2 cm in diameter [9]. In our series, four of the five cases involved tumors <1 cm, with no evidence of metastasis, and these were appropriately managed with appendectomy alone. One case (Case 3) had a tumor measuring 1.7 cm with mesoappendiceal invasion, necessitating additional surgical intervention due to the higher risk of metastasis. The management of tumors measuring between 1 and 2 cm remains controversial. Both the North American Neuroendocrine Tumor Society and the European Neuroendocrine Tumor Society guidelines recommend a conservative approach for tumors smaller than 1 cm and right hemicolectomy for tumors larger than 2 cm [15].

However, for tumors within the 1–2 cm range, optimal management remains debated. Several studies have identified high-risk features that may warrant right hemicolectomy or closer follow-up, including mesoappendiceal invasion >3 mm, lymphovascular or perineural invasion, tumor location at the base of the appendix, and a Ki-67 index >2% [15–17]. In cases with these risk factors, regular monitoring is essential to detect potential lymph node dissemination.

Histopathologically, all tumors in our series were well-differentiated neuroendocrine tumors (WHO grade I), with synaptophysin positivity in all cases but variable chromogranin staining. While chromogranin A (CgA) is a widely used biomarker for neuroendocrine tumors, its role in diagnosing ANETs is limited, particularly in early-stage tumors. Studies suggest that CgA may be more useful in detecting **metastatic or recurrent** ANETs rather than localized disease, and its levels can be affected by factors such as proton pump inhibitor use and renal function [6, 18, 19]. Additionally, two cases (Cases 3 and 5) exhibited subserosal or mesoappendiceal invasion, along with lymphovascular and perineural invasion—features associated with a more aggressive disease course and necessitating more extensive surgical management.

Although ANETs are often discovered incidentally during appendectomies, colonoscopy can play a critical role in detecting these tumors, particularly in patients with recurrent or atypical appendiceal symptoms [20]. Colonoscopy offers the advantage of identifying tumors that might be missed by imaging alone. In cases of complicated appendicitis or when histopathological examination reveals an ANET, colonoscopy should be considered to rule out synchronous tumors [21].

For patients with tumors larger than 1 cm, particularly those with risk factors such as mesoappendiceal invasion or vascular infiltration, follow-up is essential to monitor for recurrence or metastasis [22]. Routine imaging and tumor marker evaluation, combined with colonoscopy, may be incorporated into long-term surveillance plans. For patients who undergo right hemicolectomy without additional risk factors, follow-up may not be necessary; however, this decision should be individualized based on the tumor’s characteristics and the patient’s risk profile [21].

## Conclusions

This study highlights the challenges associated with the diagnosis and management of ANETs. Although the incidence of ANETs is relatively low and most cases involve small, well-differentiated tumors with favorable prognoses, larger-scale studies are needed to improve assessment and provide more robust recommendations. Additionally, future studies should explore the optimization of CT protocols and image analysis, particularly with the integration of AI, to enhance the detection of ANETs.

Table 1 provides a summary of the clinical characteristics, surgical management, histopathological findings, and outcomes of the five cases described above.

**Table 1:** A summary of the five cases of neuroendocrine tumors of the appendix.

**N**	**Age/Sex**	**Symptoms duration (days**)	**WBC count**	**Pre-op CT**	**Tx.**	**Grade of diff.**	**Localization**	**Invasion depth**	**LV/P invasion**	**Size (cm**)	**Mitosis/2 mm2**	**Ki67**	**Margin**	**pTNM**	**Post op scope**
1	28 M	1	12.9	-ve	OA	Well, G1	Distal half	Into mascularis propria and not reaching subserosa	-ve	0.3	0	<1%	-ve	T1NxMx	NA
2	37 M	1	17.3	-ve	OA	Well, G1	Proximal half	Mainly submucosal and extend to muscularis propria	-ve	0.3	0	<1%	-ve	T1NxMx	NA
3	43 M	2	11.5	-ve	Ileocecal resection	Well, G1	Distal half	The subserosa and mesoappendix	+ve	1.7	<2	<3%	-ve	T3NxMx	-ve
4	36F	4	24	-ve	OA then Rt. Lap. Hemi.	Well, G1	Distal half	Into muscularis propria into meso-appendiceal fat, without involving the visceral peritoneum	-ve	0.7	<2	NA	-ve	T3NxMx	-ve
5	30 M	1	16.1	-ve	OA	Well, G1	Distal half	Into subserosa and visceral peritoneum	+ve	0.7	<2	<2%	-ve	T3NxMx	-ve

N: number, Dx: diagnosis; M: male; F: female; Sx.: symptoms; G: Grade, NA: not applicable; Tx.: treatment; OA: open appendectomy; LA: laparoscopic appendectomy; Rt: Right; Lap. Hemi: laparoscopic hemicolectomy; CT: computed tomography scan of the abdomen; WBC: white blood cell count (4-11x10^9^/L), LV: lymphovascular, P: perineural, pTNM: pathological staging (p) the extent of the tumor (T), extent of spread to the lymph nodes (N), and presence of metastasis (M).
